# Development and validation of clinical implementation methods for patient-reported outcomes in Japanese multi-center palliative care units

**DOI:** 10.1186/s41687-024-00730-y

**Published:** 2024-05-14

**Authors:** Nao Ito, Azusa Sato, Kana Takeuchi, Tomoko Shigeno, Hiroko Sasaki, Maho Aoyama, Mitsunori Miyashita

**Affiliations:** 1 Health Sciences, Department of Palliative Nursing, Tohoku University Graduate School of Medicine, Miyagi, Japan; 2 Iwate Medical University School of Nursing, Iwate, Japan; 3 Department of Nursing, Hikarigaoka Spellman Hospital, Miyagi, Japan; 4 Department of Nursing, Iwate Medelical University Hospital, Iwate, Japan; 5 Department of Nursing, Sanyudo Hospital, Yamagata, Japan; 6 Depart of Nursing, Omagari Kousei Medical Center, Akita, Japan

**Keywords:** Palliative care, Patient-reported outcomes, Patient-reported outcome measures, Implementation

## Abstract

**Background:**

Patient-Reported Outcomes (PROs) are recommended for use in clinical oncology. However, they are not routinely used in professional palliative care practices in Japan. The reasons include both patient and healthcare provider factors and the implementation of PROs. This study aimed to develop and validate clinical implementation methods for PROs in Japanese palliative care units.

**Methods:**

The Consolidated Framework for Implementation Research (CFIR) was conducted with four palliative care units in Japan. The study was conducted in six steps: unit assessment, development and implementation of a PRO implementation plan, PRO post-implementation survey and analysis of its utilization, a review of the PRO implementation process, creation of a PRO implementation method in a palliative care unit, and use and verification of the implementation method. Steps 1–5 were the development phase, and step 6 was the verification phase.

**Results:**

Interviews were conducted with healthcare providers prior to PRO implementation. Intervention characteristics, patient needs in the palliative care unit, and factors related to the organization were identified as barriers. The implementation plan was developed, and the core members were selected. The implementation procedures were created in the above mentioned steps. PROs were used in the palliative care units. The same was true in the validation phase.

**Conclusions:**

This study guided PROs in specialized palliative care unit in a clinical setting. The method was developed and validated for the implementation of PROs in the palliative care unit. In the PRO implementation process, it was important to assess the unit, address the barriers to implementation, and reduce the burden on healthcare providers. Furthermore, healthcare providers had to be supported by the champion, a person responsible for the implementation of PROs in the palliative care unit.

**Supplementary Information:**

The online version contains supplementary material available at 10.1186/s41687-024-00730-y.

## Background

Patient-Reported Outcomes (PROs) are useful for patient-centered care and recommended for clinical use [1–3]. Their use in clinical oncology improves patient-provider communication, patient assessment, satisfaction, care by identifying patient symptoms [4, 5]. Furthermore, their routine use during chemotherapy improves patients’ quality of life and prolongs their survival [6].

However, barriers to PRO implementation exist in palliative care practice. In a survey with hospice patients, 57% required assistance in completing the Patient-Reported Outcome Measure (PROM) due to the severity of their symptoms and cognitive decline caused by the worsening of their disease [7]. Furthermore, barriers due to time constraints imposed by PROs, the inability of healthcare providers to respond to the provided information, and lack of training in PROs were also reported as barriers [8–10]. Given these barriers, ways to appropriately implement PROs in specialized palliative care remains unclear [11].

In Japan, palliative care specialists provide care in palliative care units, hospital-based palliative care teams, and home care facilities when alleviating a patient’s suffering is difficult through regular medical treatment and care. This is called specialized palliative care, which is palliative care for patients with more complex needs and serious and complicated suffering. Hence, collecting the patient’s information is necessary to assess their suffering and needs [12]. However, a survey on PROs use in specialized palliative care in Japan found that only 11% were used in palliative care units, mostly for screening at admission. In addition, limited were used routinely [13]. The reasons for this included patient factors, such as severity of symptoms and cognitive decline, and healthcare providers’ factors, such as the time required and burden. Hence, research is required on the implementation of PROs.

In implementation, a series of processes are followed to incorporate evidence-based interventions for use within an organization. Barriers and facilitators affect the outcomes [14, 15]. In addition, facilitators include the implementation of PROs tailored to organizational and patient characteristics [16, 17]. Therefore, to integrate the use of PROs into palliative care, identifying the barriers to PRO implementation and developing implementation methods to overcome them are essential. Determinants of implementation consist of barriers and facilitators, such as behavioral changes among healthcare providers, adherence to guidelines, and influence outcomes. Hence, identifying the barriers and facilitators prior to implementation is important [15].

This study aimed to develop and validate a method for the implementation of PROs in palliative care units in Japan.

## Methods

**Fig. 1:**
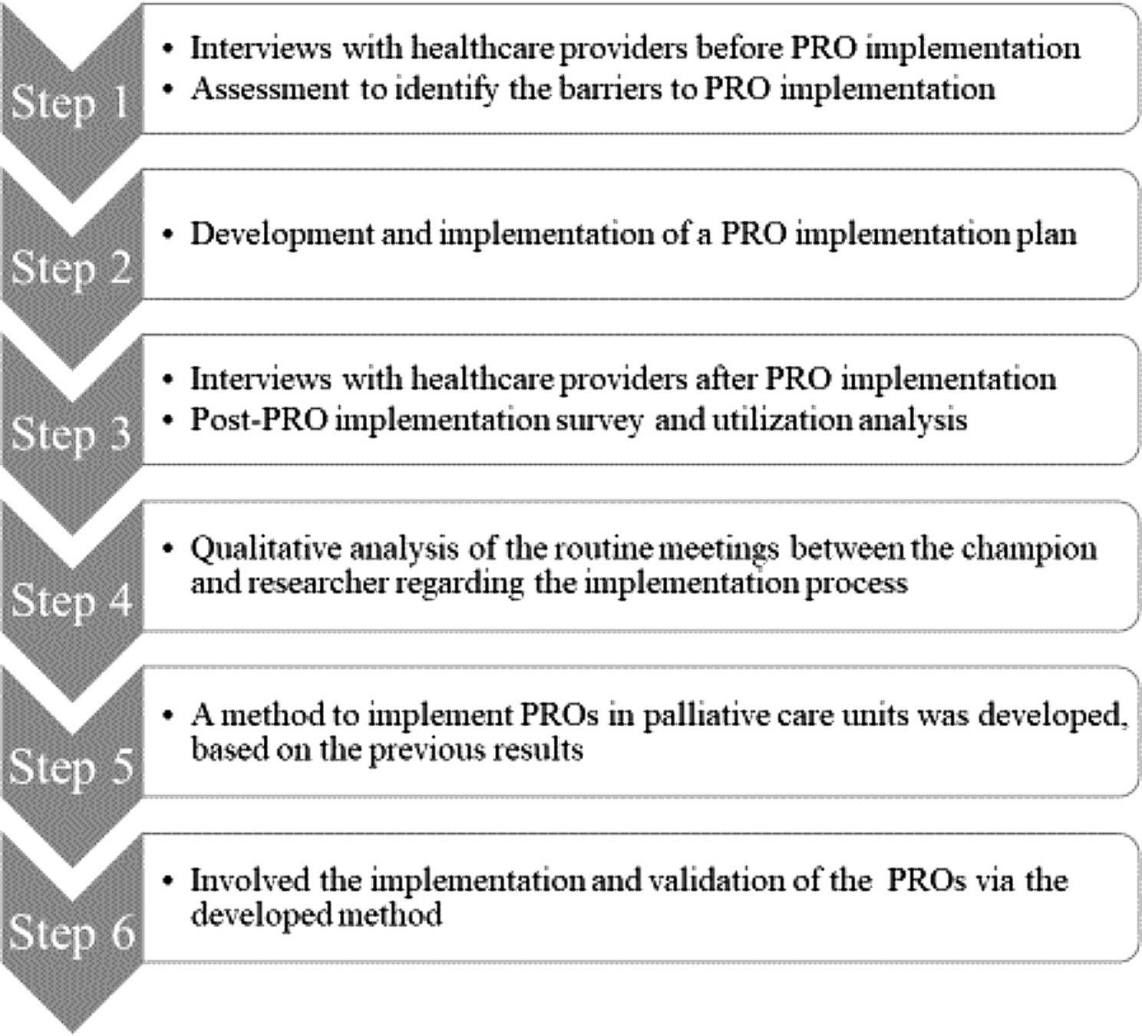
Six steps of the PRO implementing study in Japanese palliative care units

This multicenter implementation study was conducted in palliative care units for PRO implementation. The study was conducted in six steps with reference to the Consolidated Framework for Implementation Research (CFIR) [14, 15, 18, 19]. Sequentially, the steps included a unit assessment to identify the barriers to PRO implementation, planning and implementation, post-implementation survey and analysis of its utilization, a review of the PRO implementation process, creation of a PRO implementation method for palliative care units, and its use and verification. Steps 1–5 were the development phase, and step 6 was the validation phase (Fig. 1).

The person responsible for the implementation of PROs in the palliative care unit was identified as the champion. The champion was the individual who overcame any apathy or resistance that arose within the organization due to the intervention and was committed to support and complete it [11, 20]. In this study, the champion was central to the implementation of the PROs from the preparation phase.

### Settings and participants

In total, four Japanese palliative care units were recruited to participate in the PRO. Of these, three were in the development phase and one in the validation phase. Eligibility criteria for providers were that they provided palliative care and consented to participate. Eligibility criteria for patients who used the PRO were that they were admitted to a participating palliative care unit, were able to use the PRO (with assistance is required), consented to participate, aged 20 years or older, and able to speak Japanese. Patients were excluded if they had impairments or were mentally unstable.

### Research procedures (Fig. 1)

In Step 1, interviews were conducted with healthcare providers to assess the units prior to PRO implementation. The interviews enquired regarding the implementation of PROs in palliative care units: “How do you assess the patient’s distress?,” “How do you link the results of the assessment to care?,” “What do you feel are the issues with the current assessment method and what would you like to improve?,” “Is it possible to implement PROs in your unit and what methods would be feasible for you to use?,” and “What methods would be feasible to incorporate PROs in the units?” Although focus group interviews were planned, individual interviews were conducted in facilities where group interviews were difficult due to the COVID-19 endemic. Interviews were conducted online or in person by a researcher (NI), recorded, and transcribed verbatim.

Step 2 was based on the results of the analysis. Subsequently, a PRO implementation plan was developed and implemented to overcome barriers and ensure smooth implementation. The PROMs used were reviewed by the champions and researchers at each site during the preparation phase and explained to the patients before use. In total, 20 patients were admitted in each facility. Of the three development-phase units, two selected the Integrated Palliative Care Outcome Scale (IPOS). Its Japanese version has been assessed for reliability and validity among patients with cancer [21]. The other unit used the Japanese version of the MD Anderson Symptom Inventory (MDASI-J), which has also been validated and was reliable [22, 23].

Step 3 involved interviews with healthcare providers after the PROs were implemented, and their use was verified via medical records. Interviews after PRO implementation asked how they used PROs, their thoughts, whether they thought it was possible to continue utilize PROs, and in what ways. The PRO implementation rate was calculated as the percentage of PROs used among all patients admitted to the palliative care unit during the study period who met the eligibility criteria. PRO utilization was defined as whether PRO results were shared at conferences, care plans were developed or modified, PROs were used, and treatment and care were implemented from medical records. In addition to the patient’s PRO score, patient information, such as age, sex, diagnosis, Performance Status (PS) at the time of admission, duration of their admission, outcome, and reasons for PRO discontinuation, were also obtained.

Step 4 was a qualitative analysis of the routine meetings between the champion and researcher regarding the implementation process. In step 5, a method to implement PROs in palliative care units was developed, based on the previous results. Step 6 involved the implementation and validation of the PROs via the developed method. The IPOS was used during the validation phase.

The survey period for each facility was approximately six months, and all data were collected from November 2020 to March 2022.

### Data analysis

Interviews with healthcare providers were reviewed via thematic analysis [24]. The analysis was reviewed until a consensus was reached among the experts in palliative care research (NI and MM).

We performed a Wilcoxon signed-rank sum test for each PRO score. All analyses were performed using JMP Pro16 data analysis software (SAS Institute, Cary, NC, USA), with a significance level of 5%.

## Results

### Step 1: Assessment to identify the barriers to PRO implementation

A total of 39 healthcare providers were interviewed before PRO implementation at the three facilities (Table 1). Barriers to PRO implementation were analyzed from the interview data and included “selection of individual patients” and “burden on healthcare providers.” Regarding intervention characteristics, “difficulties in self-evaluation among older patients and patients with cognitive decline” and “characteristics of patients who did not wish to self-evaluate” were identified. Regarding patients’ needs, “palliative care unit organization,” “routine evaluation by healthcare providers,” “challenges with existing evaluation methods,” and “culture of unwillingness to change” were identified. We also identified the categories of “challenges in listening to patients” and “concerns regarding the use of PROMs” related to the individual characteristics of palliative care unit healthcare providers (Appendix 1).

**Table 1:** Facilities’ background

Facility background	Development phase (Three facilities)		Verification phase (One facility)	
Number of beds	20, 25, 12		13	
Average number of days in hospital (days)	34, 26, 13		17	
Return-to-home ratio (%)	4, 8, 64		30	
Healthcare provider interview participants	Pre implementation	Post implementation	Pre implementation	Post implementation
Number of participants (persons)	39	38	8	10
	Group 8G Individuals 13	Group 6G Individual 16	Group 2G	Group 2G
Participant occupation				
Physician	2	3	1	2
RN	36	35	7	8
Music therapist	1			
Interview duration (minutes, mean ± SD)	21.4 ± 9.3	17.0 ± 6.2	20.0 ± 0.0	27.5 ± 3.5

### Step 2: Development and implementation of a PRO implementation plan

The PRO implementation plan was designed to address the identified barriers. First, core members were selected to facilitate the implementation of PROs in their unit. Patient selection criteria were developed for the first time, as well as procedures to be shared at the conference for problems after PRO implementation. Assessment timings were set (on the day of admission, third day of admission, first week of admission, and every week thereafter) and study sessions were held for healthcare providers. Throughout the entire study process, champions shared information with unit managers and physicians and continued educational involvement and support for medical personnel.

### Step 3: Post-PRO implementation survey and utilization analysis

In total, 38 healthcare providers were interviewed after the implementation of PRO (Table 1). Table 2 presents the results of the interview data analysis. As implementation outcomes, the effects of PRO implementation were identified as “facilitation of patient-healthcare provider communication,” “awareness of patients’ thoughts and vexations,” and “patient-centered care.”

**Table 2:** Effectiveness of implementing PROs in Japanese palliative care units: results from interviews with healthcare providers

Category	Subcategory
Patients and healthcare providers Facilitating communication	Opportunities for communication between patients and healthcare providers.
	PROMs become a communication tool for patients and healthcare providers
	Assessment at the time of admission is valid
	Can be heard by PROMs
	PROMs make it clear what to ask
Awareness of patient’s thoughts and pains	Be aware of the patient’s thoughts and vexations
	A comprehensive view of the patient
	Realize that there is a disconnect between patient and health care provider evaluations
	Realize the importance of patient assessment
Patient-centered care	Practice patient-centered care
	Finding direction of care
	Utilize for patient-centered nursing planning
	Transforms into an individualized record
PRO’s use at conferences	Share evaluations at conferences

### PRO implementation rate and utilization

During the study period, 125 patients were admitted to the palliative care unit and 59 (47%), met the eligibility criteria, responded to any part of the PROM on the day of their admission. Of these, 47 also responded on the third day of admission, and 35 on the seventh day.

Of the patients who met the eligibility criteria for PRO use, 100% completed it on the inpatient day. For PRO utilization, the three development phase sites had 68% conference, 61% care planning and modification, and 68% treatment and care implementation on the inpatient day. Furthermore, on day 7 these were 69%, 69%, and 72%, respectively. In the assessment on the admission day, day 3 and day 7, day 3 was the least utilized, with 38% planning and modification of the care plan.

### Change over time in PROs in patients

In the two facilities that used the IPOS in the development phase, there was no significant difference in patient scores for each item between admission day and day 3. Regarding each item for patients from admission day to day 7, there were significant differences. Constipation and sleepiness worsened from 0.73 on admission day to 1.32 on day 7 (*p* = 0.009) and 0.59 on admission day to 1.23 on day 3 (*p* = 0.002), respectively. For patients with data from the day of admission to day 14, significant differences were found for fatigue and sleepiness, which worsened from 0.33 on day 3 to 1.89 on day 14 (*p* = 0.008) and 0.33 on day 3 to 1.44 on day 14 (*p* = 0.008), respectively. In the one facility that used the MDASI, there were no significant differences in patient scores.

### Step 4: Reflection on the PRO implementation process

During the study period, the champion and researcher met monthly to reflect on the implementation process. A qualitative analysis of the contents revealed that the preparation period was spent clarifying issues and setting goals in the units. The first month of implementation was spent experiencing the benefits and difficulties of PRO. Months 1–2 months and 2–3 were spent surfacing issues with establishing and utilizing PRO, and discussing the changes in conferences and care due to PRO and the changes in the units, respectively.

### Step 5: Creation of a method to implement PROs in the palliative care unit

The method to implement PROs in the palliative care unit was developed based on the results of steps 1–4 (Table 3).

**Table 3:** PRO implementation methods in the palliative care units

(Data) item	Specific methods
Preparation before implementation	Select a champion
	Select two or three core members
	Respond to questions in the absence of a champion
	Publicize the implementation of PRO as a unit initiative through announcements and electronic medical record email function
	Conduct workshops on PROs and PROMs
	If it is difficult for everyone to attend a study session, use video content
	Study sessions are held during existing meetings to minimize the time burden
	Schedule PRO evaluations and maintain records.
	Use magnets, calendars, and electronic medical record bulletin boards to keep track of patients scheduled for PRO evaluations
	Determine the format to be used
	Use of PROs on hospitalized patients
	Champion or core members use PRO on first patient and share at conferences
	Share what was good about using PRO at the conference
Selection of the patients	Basically, all patients admitted to the Palliative Care Unit
	If a patient is older or cognitively impaired and has difficulty answering the question, replace it with words that are easier to understand.
	Consider having a medical provider evaluate a patient with delirium or unstable status who is finds it difficult to answer
	Palliative care units do not overstep their bounds as some patients have difficulty with PRO as they progress
	The first time a PRO is used in a unit, it should be a patient with a scheduled admission and no cognitive decline or delirium
	If the patient does not want or refuses to have a PRO, do not force the patient to have a medical evaluation
Timing of the evaluation	Use PRO on the day of admission or the day after admission
	One week after the initial evaluation, and every week thereafter
	If a prolonged hospitalization is anticipated, the evaluation after one week should be spaced according to the patient’s condition
	Shift the evaluation date to the next day or end of the week depending on the patient’s condition and unit’s situation
How to listen	Explain the purpose of PRO to the patient
	Ask in a clerical, one-question-at-a-time tone
	Ask the reasons for the patient’s evaluation
	Basically, ask all the items in the PROM; however, ask regarding some items if you do not want to answer
	You may focus on the items you were concerned about last time and ask
	When the patient’s evaluation and the healthcare provider’s evaluation are mixed, record them so that it is clear who did the evaluation for each
	Ask regarding psychosocial items only after confirming that you are allowed in consideration of their impact on the patient
	Tell them that they can stop halfway through if it gets too hard
	Healthcare providers should understand that they may not be able to answer immediately after being given bad news or during a worsening condition or
	If the patient is able to fill out the PROM on their own, have them fill it out; however, be sure to check the results and listen to what they have to say
	If you are not comfortable using PROMs and being asked questions, ask about the items in conversation and care
	In the second and subsequent sessions, ask regarding items that were of concern in the previous session and compare the results
	If the patient cannot answer numerically, devise a way
	Use a face scale or visibility chart
	If the conversation is too long and interferes with work, tell them in advance how long you will be gone before asking them
Utilization of PRO Results	PRO results are shared as much as possible on the same day or at next day’s conference
	Develop and implement a care plan based on the PRO results
	Record in the medical record any items of concern or patient comments from the PRO results.
What to do if you have trouble with an evaluation	When in doubt, use a large number to evaluate a patient’s concern or distress
	When there is a discrepancy in the evaluation between the patient and healthcare provider or when there is confusion in the evaluation, share the information at the conference
Role of the Champion	Maintain the evaluation schedule and speak to the person in charge until it is established
	Positive feedback is given when PROs are used and utilized in care
	Check and address any problems or concerns after PRO implementation
	Opportunities to exchange opinions after PRO implementation
	I am going to do it first, and I am going to keep asking people to do it
	Supporting medical professionals using PRO for the first time
	Coordinate workload, e.g., not assigning multiple patients scheduled for PRO evaluation

In preparation for PRO implementation, the core members who would be champions and collaborators in the unit were determined. Study sessions were held on PROs and PROMs. Ways to schedule, format, and record patients to be evaluated by PROs were determined. Furthermore, ways to implement the PROs in the unit were also determined.

All patients admitted to the palliative care unit were included; however, if the patient did not want or refused to complete a PRO, healthcare providers evaluated the patient without forcing them. If PRO was difficult for older patients or those with cognitive decline, the term was replaced with an easier-to-understand term. During PRO use, its purpose was explained to the patient at the beginning and the patient was asked not to use a question-and-answer style. The results of the evaluation were shared at a unit conference on the same or next day to discuss the care plan.

Until the PRO was established in the unit, the champion managed the evaluation days and provided ongoing support by approaching healthcare providers to check if they had any problems.

### Step 6: Utilization and validation of the implementation strategy

During the validation phase, group interviews were conducted before and after implementation (Table 1). Barriers to PRO post-implementation were identified from the interview data, and the same categories were extracted as in the development phase. Of the patients who met the eligibility criteria for PRO use, 100% used it on the day of admission. PRO utilization was 85% conference, 100% care planning and modification, and 100% treatment or care implementation. On day 3 conference utilization and treatment or care implementation was 94%. On day 7 conference was 80% and care planning and modification and implementation of treatment and care was 100%.

During the study period, 26 patients were admitted to the palliative care unit categorized for the validation phase. Of these, 20 (77%) responded to any part of the PROM on the day of admission and met the eligibility criteria. In addition, 16 responded on the third day, and 12 on the seventh day.

The validation phase site used the IPOS and found a significant difference between the day of admission and day 3. Fatigue worsened from 1.87 to 2.53 (*p* = 0.032). No significant differences were found for each item from the day of admission to day 7.

The PRO implementation method was completed, except on the third evaluation day, after the completion of all surveys.

## Discussion

This study developed and validated a method to introduce PROs into care units in specialized palliative care. The unit had to be assessed and barriers to implementation had to be addressed to overcome, which would lead to PRO retention. Introducing PROs in palliative care units was important to reduce the burden on providers. In addition, supporting providers with a champion was critical.

### Assessment and overcoming barriers to PRO implementation

The first major finding was that PRO implementation in palliative care units could be established if the units were assessed and addressed. Hence, barriers to implementation could be overcome. Barriers to PRO implementation in palliative care units were identified through interviews with healthcare providers prior to implementation at collaborating facilities who wished to implement PROs. Furthermore, the issues to be addressed during PRO implementation were identified, as in the previous study by Coffey et al., and included criteria for PRO eligibility, assessment timing, and post-assessment conference dates [16]. Furthermore, additional barriers to PRO implementation were also reported [9, 10]. We determined the method of selecting participants and timing of the assessment, which were identified as barriers to PRO implementation, and provided education to healthcare providers to facilitate smooth implementation. Therefore, after 1–2 months, PROs were proactively utilized by healthcare providers and became firmly established in the units [25].

We also identified a category of “characteristics of patients who did not wish to self-evaluate” regarding the characteristics of patients who left it up to the medical provider to introduce PROs. Owing to the history of preference for specialized in the Japanese culture, we do not believe that PROs should be used in all patients if they refused [8].

### Innovations to reduce the burden on healthcare providers through the implementation of PROs

PRO implementation in palliative care units required careful preparation and specific measures to reduce the burden on healthcare providers. The key was to create a system in which the results were utilized rather than being left unanswered. Hence, incorporating the measures listed in Table 3 into facilities will be useful.

In this study, patients eligible for PRO found it difficult to use them owing to their worsening disease status and decreased state of consciousness. Since the PROM was a structured and standardized questionnaire, it should be used by patients without changing the wording in principle [26]. However, in palliative care units, PRO may be difficult owing to the progression of the patient’s disease or decline in cognitive function. Hence, adapting the PRO to the patient’s condition by changing the wording to make it easier to answer or ask through conversation is important, as done by healthcare providers in this study [27]. However, it is necessary to use PROs with caution as they may be influenced by the emotions and experiences of those other than the patients [28].

### Role of champions in PRO implementation

Regarding the implementation of PROs in palliative care units, the importance of providing a champion support to healthcare providers was highlighted. In this study, champions provided support to healthcare providers throughout the PRO implementation process. On the day of admission, PROs were used in 100% of the patients who met the eligibility criteria. Furthermore, utilization in treatment and care resulted in PRO retention, with 68%, 72%, and 100% on the day of admission in the development phase, day 7, and validation phase, respectively [17]. Bausewein et al. successfully implemented PROs routinely in palliative care practice. In addition, the role of champions was important in Japanese palliative care units as well [29–31].

### Usefulness of the patient’s PRO

There was no significant difference in the improvement in patients’ PRO scores between the day of admission and day 3 via the IPOS or MDASI-J PROMs. This suggested that many patients admitted to palliative care units required palliative care, which made it difficult to achieve the targeted score improvement. However, PROs allowed providers to assess patient distress, which could lead to palliative care. Furthermore, Campbell et al. stated that the clinical significance of the use of PROs should be examined. Hence, it was important to evaluate PROs in palliative care units regarding score improvement and also facilitating communication between patients and healthcare providers [32].

### Limitations

This study had some limitations, First, we conducted a survey with healthcare providers. Hence, this may not reflect the effectiveness of PROs in patients. Second, this study focused on a specific palliative care unit and did not analyze the barriers to PRO implementation in the community or organization as a whole. Hence, analyzing the factors related to the community and organization as a whole is necessary to promote PROs in palliative care practice. Third, this study was conducted in a palliative care unit. Hence, the implementation of PROs in general units and homes should be considered.

## Conclusion

This study developed and validated a method to implemented PROs in care units within a specialized palliative care clinical setting. The process of implementing PROs in palliative care units involved assessing the unit and addressing the barriers to implementation, reducing the burden on healthcare providers, and championing support providers.

### Electronic supplementary material

Below is the link to the electronic supplementary material.

Appendix 1 Barriers to introducing PROs in palliative care units

## Data Availability

Data supporting the results of this study are not available to the public. However, they are available from the Division of Palliative Care Nursing at the Department of Health Sciences (Tohoku University Graduate School of Medicine) upon reasonable request to the corresponding author or the division administrators (http://www.pn.med.tohoku.ac.jp).

## Abbreviations

IPOS: Integrated Palliative care Outcome Scale
MDASI: MD Anderson Symptom Inventory
PRO: Patient-reported outcome
PROMs: Patient-reported outcome measures
