# Supplementary material for: Development and validation of clinical implementation methods for patient-reported outcomes in Japanese multi-center palliative care units
Source: J Patient Rep Outcomes. 2024 May 14;8:49. doi: 10.1186/s41687-024-00730-y (PMC11093964; doi:10.1186/s41687-024-00730-y)
Supplement: Supplementary file 1 — Appendix 1 Barriers to introducing PROs in palliative care units [file 41687_2024_730_MOESM1_ESM.docx]

| Appendix 1: Barriers to Introducing PROs in Palliative Care Units | |  |
| --- | --- | --- |
| Barrier | Category | Subcategory |
| Characteristics of the intervention | Selection of individual eligible patients | Patients who can evaluate themselves can use it |
|  |  | Eligible patients are limited. |
|  | Burden on healthcare providers | Time-consuming to ask the patient |
|  |  | Burdened because the patient hears |
|  |  | If you start without knowing, you cannot take advantage of it |
|  |  | Anxious to evaluate multiple patients (validation phase) |
|  | Limitations of evaluation in PROMs | Tools and numerical evaluations are difficult to understand. |
|  |  | There is a difference between the numbers and the patient experience. |
|  |  | No clear psychosocial PROM |
|  |  | Individuals differ in how they perceive the rating scale (validation phase) |
| Patients’ needs | Difficulties in self-assessment in older patients and patients with cognitive decline | Older patients, patients with cognitive decline and delirium have difficulty self-assessing |
|  | Difficulties with numbers and scale evaluation | Difficult to evaluate by numbers and scales |
|  | Characteristics of patients who do not wish to self-evaluate | Resistance to being asked repeatedly |
|  |  | There are patient characteristics that do not wish to self-evaluate |
|  | Patient's condition is unstable (validation phase) | High number of patients with unstable condition at the time of transfer to palliative care unit (validation phase) |
| Organizational culture of the unit | Routine medical evaluations | Observed and evaluated by a medical professional |
|  | Challenges with previous evaluation methods | Evaluation is not timely |
|  |  | Assessments are not utilized in care |
|  |  | Conference records are not utilized |
|  |  | No assessment over time (verification phase) |
|  |  |  |
|  | Experience with tools not being utilized | Experience with tools not being utilized |
|  |  | Tools are not utilized due to lack of study by evaluators |
|  | Concerns regarding PROM utilization | Concerned about asking the same thing in the PROM |
|  |  | New PROMs take time to establish |
|  | Climate unwilling to change | There is a trend that PROMs should remain in their current form |
| Individual characteristics of healthcare providers | Challenges in listening to patients | Unable to step in and ask the patient |
|  |  | There is a lack of asking patients |
|  |  | Struggling with how to ask about psychosocial aspects |
|  |  | Hesitate when to ask the patient |
|  | Concerns about PROM utilization | PROMs do not fit the patient. |
|  |  | Having to use the tool is a burden |
|  |  | Hard to see feelings and spirituality in PROMs |
|  |  | I hesitate for time to ask. |
|  | Possibility of subjective evaluation by medical professionals | Subjective evaluation by a medical professional |
|  |  | Evaluate the patient's reactions and changes |
|  | Challenges of using patient assessment | Failure to utilize patient assessments in care |
|  |  | Evaluating care using assessments is difficult |
